# GM insect pests under the Brazilian regulatory framework: development and perspectives

**DOI:** 10.1186/s12919-018-0107-z

**Published:** 2018-07-19

**Authors:** Paulo P. Andrade, Marília Andreza da Silva Ferreira, Marta Silva Muniz, Amaro de Casto Lira-Neto

**Affiliations:** 1 Centro de Saúde e Tecnologia Rural, Universidade Federal de Campina Grande, Patos, PB Brazil; 2 Departamento de Genética, Universidade Federal de Pernabuco, Recife, PE Brazil; 3 Instituto Agronômico de Pernambuco –IPA, Recife, PE Brazil

## Abstract

The emergence of new technologies for genetic modification has broadened the range of possible new products. The regulations of many countries that could benefit from these new products may not be prepared to assess risks and enable science-based decision-making. This is especially acute in the case of genetically modified insects with potential use in public health and agriculture. Modifications of the regulatory framework, sometimes necessary to allow a proper risk assessment of products from new technologies, are strongly influenced by political decisions derived from the balance of power and interest among stakeholders. This article discusses the genesis of the Brazilian regulatory framework, its applicability for the risk assessment of genetically modified insects and the scenarios that have shaped the two biosafety laws that established the basis for the use of modern biotechnology in the country. It is concluded that, for the adoption of the new technologies, it is important to carefully navigate the political tensions by seeking the engagement and empowerment of stakeholders supporting science-based decision-making in order to gather the necessary support for adoption of risk assessment as the basis for final decisions, allowing the use of new technologies.

## The structure and function of the Brazilian National Biosafety System

Like many other countries, Brazil has adopted a technology-based approach to regulate GMOs [1]. In 1995, the first Biosafety Law created a centralized agency – the National Biosafety Technical Committee (CTNBio) - to assess GMO risks and to decide on a scientific basis if a GMO can be used in field (containment) experiments or commercially released. CTNBio also issues certificates on biosafety, essential to perform any activity related to GMOs in public and private institutions. Due to many conflicts with the existing legal framework, the law was considered inadequate and was substituted in 2005 by Law 11,105/2005 (http://bch.biodiv.org/database/attachedfile.aspx?id=1601), which removed the conflicts and empowered CTNBio as the central body for biosafety regulation. Its main responsibilities are outlined in Table 1 below.

**Table 1:** Main responsibilities and functions of the National Biosafety Technical Committee (CTNBio)

• Issues authorizations necessary for research with GMOs and their derivatives in the country (Biosafety Quality Certificates, authorizations for releases in the environment and others); • Issues technical opinions on the commercial releases of GMOs, which are binding on other Government agencies; • Provides technical advisory and advisory support to the CNBS – National Biosafety Council in the formulation of the National Biosafety Policy of GMOs and their derivatives. • CTNBio deliberates, in a final and definitive instance, on which cases the activity is potentially or effectively causing environment degradation or human/animal health harm.	

CTNBio is an entity composed of representatives from all five relevant ministries (http://www.planalto.gov.br/ccivil_03/decreto/1995/D1520.htm) and its decisions, primarily based on risk assessment, cannot be overturned by any other instance or legislative body except the National Biosafety Council (CNBS), composed of 11 State Ministers. Even this Council may only argue based on social or economic issues directly linked to the intended commercial use of the new GM product. However, if new hazards or damages are identified by any third party, they can be reported to CTNBio, which must reassess the product and, if non-negligible risks are identified, act accordingly.

**Fig. 1:**
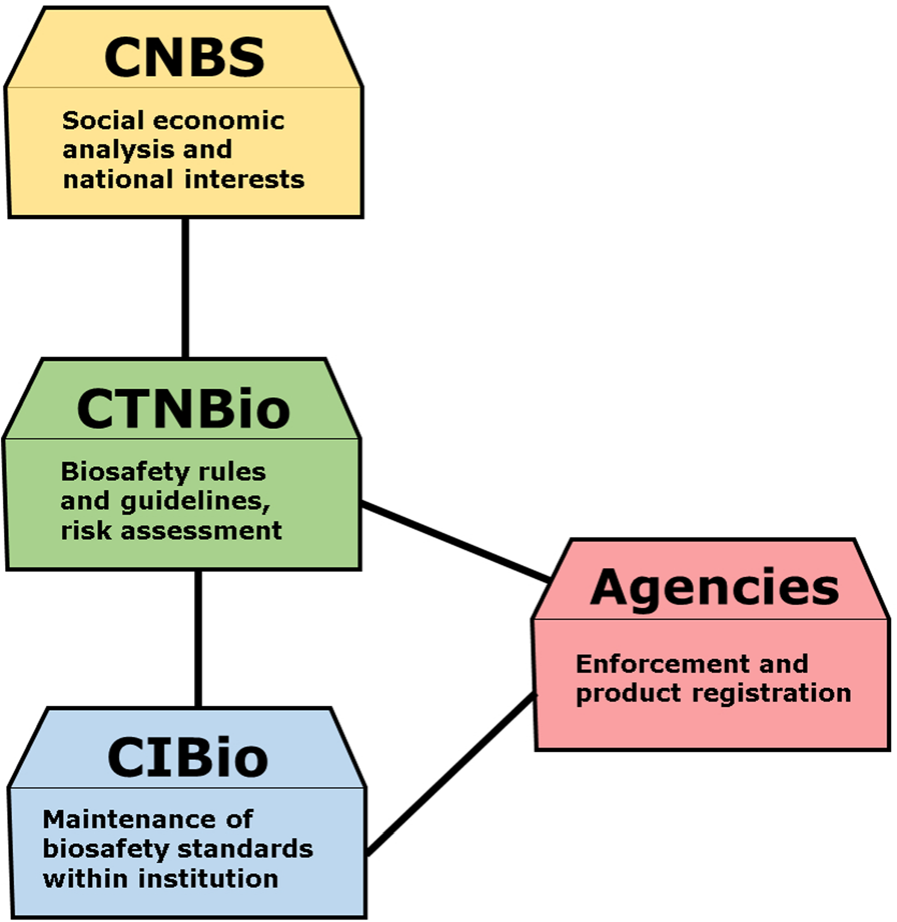
Relations between the main components involved in the Brazilian regulatory system. The whole system is based on the decisions of CTNBio, a technical-scientific body. The Commission assesses risks associated to GMO, authorizes field trials and, at the end of the process, may grant permission for its commercialization. Only biosafety and science-based issues are taken into account in the risk assessment that supports the technical recommendation. In turn, the CNBS, which is hierarchically superior, has the power to veto the marketing of genetically modified products based on social or economic considerations. CIBios are commissions within institutions / companies working with GMOs and are directly subordinated to CTNBio, which grants them a certificate of biosafety-CQB, which is a license given by CTNBio to the requesting institution to carry activities involving GMOs and their derivatives. Communication must be constant between them. Another important player is the set of agencies responsible for Registration and Inspection/Enforcement (ANVISA – the National Health Surveillance Agency, IBAMA - Brazilian Institute of Environment and MAPA – Ministry of Agriculture, Livestock and Supply). They inspect GMO field releases and register products once the commercial release is granted by CTNBio

Both the Council (CNBS) and the Committee (CTNBio) integrate a network including regulation/enforcement agencies and local institutional Internal Biosafety Committees – CIBio (Fig. 1). Moreover, as a Party to the Cartagena Protocol on Biosafety, Brazil has also to comply with a set of specific requirements on transboundary GMO movement.

## A brief history of the stakeholder scenarios before and after the enactments of the first and second biosafety Laws

Brazil was one of the first countries to embrace agricultural biotechnology. Following a then prevailing international trend of regulating the technology, instead of the product, in 1995 the country produced a new specific law on the use of transgenic organisms and adapted the existing legal framework to assess GMO risks and pave the road for the adoption of agricultural biotechnology.

Regulators and scientists then faced the difficult task of weaving the first GMO regulatory framework in a complex scenario, with very vocal stakeholders against biotechnology, a populist government and a general lack of experience in dealing with GMOs [2]. The Brazilian Agricultural Research Corporation - EMBRAPA, a state-owned research corporation, and other Brazilian private or public companies, as well as the transnational biotech giants, were actively engaged in the development of the new law, but resistance from international environmental NGOs and social movements, connected to landless “campesinos” and other opponents, were able to weaken the law, which slightly changed the existing laws as not to conflict with the Biotechnology law (issued 1995; http://www.planalto.gov.br/ccivil_03/leis/L8974.htm). This, in turn, created unsurmountable conflicts of authority [3], mainly between the Institute of Environment and Natural Resources (IBAMA), an uncompromising defender of the environment and clearly against GMOs, and the National Biosafety Committee (CTNBio), in charge of GMO risk assessment, but accused of having excessive links with the private sector and not having independence in their risk assessment.

By exploring the existing legal conflicts in the regulatory framework, the GMO opposition was able to halt the release of the sole GMO approved for commercial release by CTNBio in 1998, a genetically modified soybean variety, and establish a *de facto* moratorium, disqualifying CTNBio’s decisions and requiring an overly complex and onerous process for the approval of commercial releases of new GMOs [3, 4].

**Fig. 2:**
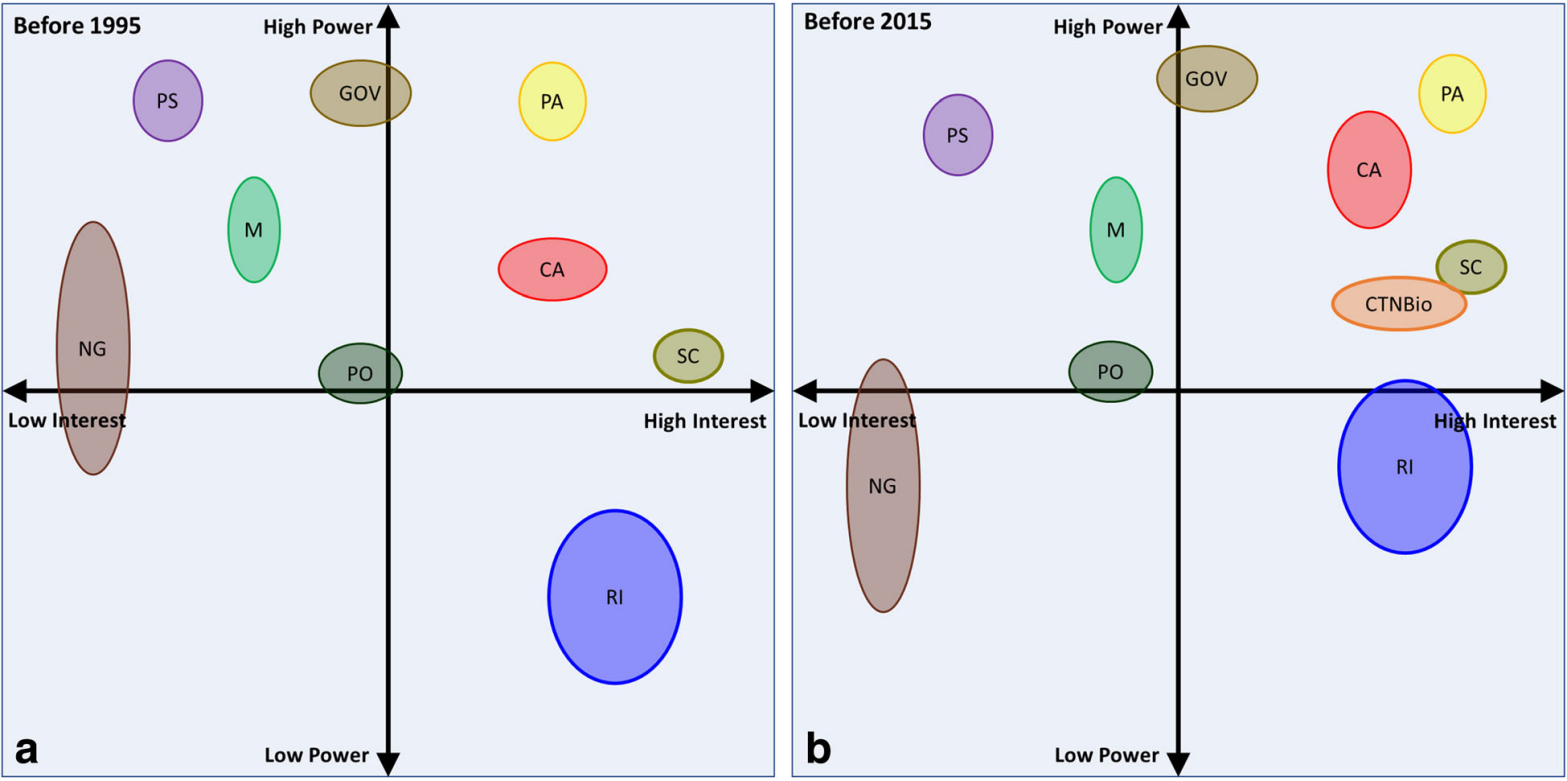
Positioning of main stakeholders influencing the construction of Brazil’s 1995 and 2005 biotechnology laws. The x and y axes represent the level of interest and power of influence, respectively, in shaping the Brazilian regulatory framework. **a** depicts the scenario of the discussions that preceded the creation of Law 8.974 / 1995 and (**b**) the positioning of the different stakeholders in shaping the present law 11.105 / 2005. When comparing these two scenarios, it is worth noting the increase in the power of influence of the agribusiness sector and of research institutions, together with the increase of interest of the Brazilian government and agribusiness politicians. This was mainly due to the clamor of large commodity planters, such as soybeans, who saw neighboring countries like Argentina increase their productivity using herbicide-resistant GMO seeds. In this context, groups opposed to the adoption of this technology, such as NGOs and politicians linked to social movements, who had a significant influence on decision making in scenario A, lost strength in scenario B and influenced media to a lesser extent. Both the public and the media began to perceive the benefits of technology for an agricultural country like Brazil, as well as the hypothetical risks. However, public opinion proved to be more resistant to a significant change, possibly as a consequence of the intense NGOs and social movements influence during the shaping of the first law. CTNBio appears only at moment B because it was created with the law 8.974 / 1995. Seed companies (SC); Research institutes (RI); Politicians aligned to agribusiness (PA); Politicians aligned to social movements (PS); Public opinion (PO); Commodity agroindustry (CA); Government (GOV); Media (M); NGOs and Social Movements (NG); National Biosafety Technical Committee (CTNBio)

A new strong stakeholder was decisive in reversing the moratorium: the soybean growers of Southern Brazil. As depicted in the stakeholder maps from Fig. 2, the Soybean Associations already had some relevance in the scenario prior to the enactment of the first Biosafety Law due to the fast development of the soybean sector in Brazil in the 90’s. In neighboring Argentina, growers were experiencing increased profit due to the early adoption of transgenic soybeans and the Brazilian soybean farmers were falling behind and losing competitiveness. As a legal reversal of the moratorium was very unlikely, a large group of growers decided to smuggle seeds from Argentina, which has a very similar climate and soil characteristics. The illegal planting probably started as early as 1999 and in the 2003 harvest there were already more than 10 million tons of transgenic soybeans in the silos [5]. In the meantime, there was a growing consensus among a large group of stakeholders that the confused legal framework was causing enormous scientific and technological damage to the country, as well as leading to significant losses of economic opportunities in the field of biotechnology, both agricultural and industrial [2].

By 2004, the legal conflicts in the regulatory framework were considered to be unsurmountable [4]. The Federal Government also had to deal with the illegal soybean harvests and the country’s debut as Party to the Cartagena Protocol, which opened for signature in 2000. Brazil ratified the Protocol late in 2003, which came into force in February 2004 (https://bch.cbd.int/about/countryprofile.shtml?country=br). The progressively larger adoption of GM plants, the lack of any evidence of negative environmental impact or health damage due to the planting and consumption of GM plants and a wider and more influential participation of the scientific society in the debates around GMOs and stem cells [6] contributed to a relative power reduction among opposition stakeholders. The changing scenario prompted the Brazilian Government in 2005 to issue the new Biosafety Law that is now in force, and to rewrite or revoke all other conflicting laws and decrees as to resolve the previous legal imbroglio. Under the new law, CTNBio has essentially the same duties, but has more power, because its decisions are sovereign and legally binding.

The adoption of both agro- and industrial biotechnology has progressed immensely ever since in Brazil, with the adoption of GM products for many different applications, such as cellulose production, second-generation ethanolic fermentation, vaccines, diagnostics and recently, vector control. Indeed, the transgenic OX513A *Aedes aegypti* was considered safe by CTNBio in 2014 [7] and is currently under efficiency assessment in the State of São Paulo.

## Transgenic insects under the Brazilian regulatory framework

**Fig. 3:**
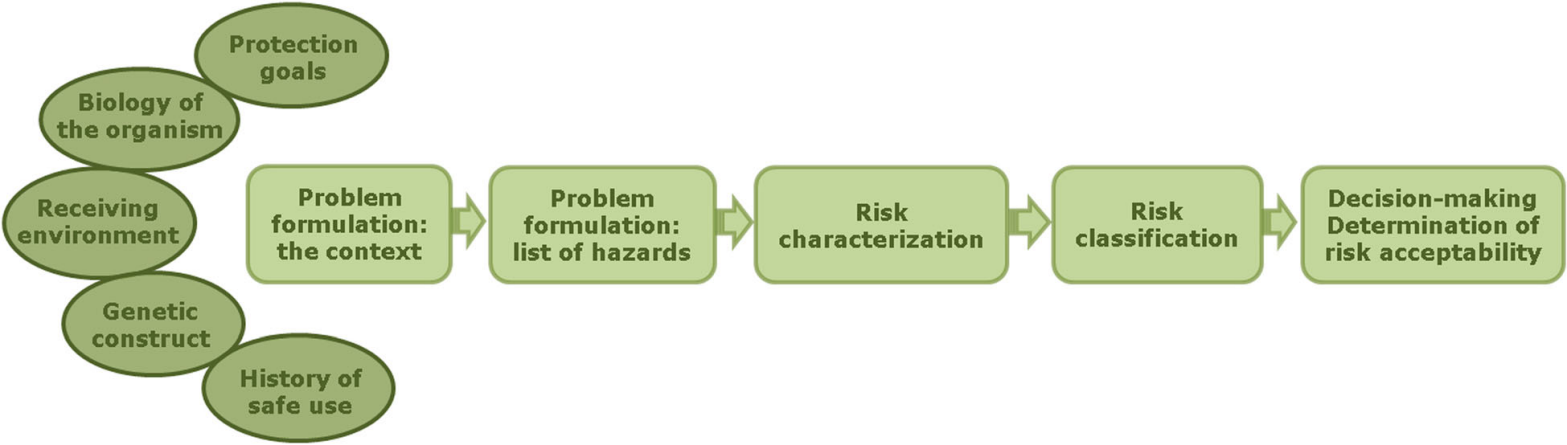
Diagram presenting the methodology of risk assessment of GMOs, with its five stages. Ellipses on the left represent the information needed to define the context where the GMO will be used

The step-by-step approach to GMO risk assessment was developed in the last 20 years, building on the experience of many specialists, and is largely accepted [8–10]. The five steps of a complete environmental risk assessment followed at CTNBio encompass a large set of information needed to establish the context in which the GMO will be used, either in containment, confinement or in large scale, unconfined releases. They also establish the procedures to classify the probabilities of a hazard to materialize into harm and how to classify the extent of the consequences (or harm). Finally, the approach also allows a logical and systematic use of likelihoods of exposure to the GMO and harm extension to assess risks for each conceivable or perceived hazard (Fig. 3).

Although transgenic plants were the main subjects of the first risk assessments, animals, bacteria, protozoa, fungi and viruses were also regarded as potential organisms to be assessed, and this allowed the accommodation of specific questions relative to different taxa in the broad risk assessment procedure. In the Brazilian regulatory framework, Normative Resolution nr. 5 (http://ctnbio.mcti.gov.br/resolucoes-normativas) regulates the unconfined commercial release of GMOs and is structured as a primary general part, followed by a set of annexes with specific questions needed to assess food and feed safety (Annex III) and environmental risks (Annex IV). This last annex has specific questions aimed towards organisms used for biological control (item E) and invertebrates (item I). The use of question lists, while providing some legal certainty to the applicant and helping in the identification of possible hazards, rigidifies the risk assessment. Aware of this limitation, CTNBio exercises its right to add appropriate questions for each new risk assessment and, likewise, exempts the applicant from responding to questions that do not apply to the GMO being evaluated.

When assessing OX513A *Aedes aegypti* risks in confined trials back in 2011, CTNBio had to deal with an important new question, i.e., the dispersal of released male mosquitoes out of the experimental area. Although GM plants can also disperse in the environment, regular control measures can effectively confine the plants to the experimental area, but there was no immediate, obvious way of controlling mosquito dispersal. Both normative resolutions (NRs. 6 and 8) regulating confined releases were of little help with this concern. However, as the genetic construct inserted in this GM mosquito determines its death and that of its offspring, the commission considered the dispersion to be very limited due to the short life span of the insects. Although a few insects escape the lethal mechanism in the first generation, they are very unlikely to do so in the next generation and the chances of dispersion are very low, even in the absence of any physical barrier. A similar approach may be used to any self-limiting GM insect, both for vector or for pest control.

Specific questions from Annex IV were of some help to derive possible hazards associated with the transgenic *Aedes* in the unconfined release risk assessment phase, but most plausible hazards came from the regular assessment of the context, as determined by the application of the same risk assessment guidelines used for other GMOs. Similarly, plausible hazards from other GM insects will possibly derive in a straightforward way from the application of the regular risk assessment guidelines.

As with the discussion surrounding the approval of planned releases and the commercial release of the GM *Aedes*, it is likely that non-biosafety issues will be brought to the forefront by stakeholders opposed to other GM insects (e.g., technology dependency, less expensive solutions, natural resistance to the genetic constraint, etc.). This is a common strategy in many fora and a well-delineated regulatory framework can avoid mixing issues relevant to risk assessment with those important in other contexts (economic, social, etc.). Brazilian legislation requires that the risk assessment of GMOs be the exclusive attribution of CTNBio, while economic and social issues must be addressed by the various ministries that will register and supervise the use of the new product or by the National Biosafety Council (CNBS). This strategic separation prevents CTNBio from having to discuss and take into account aspects that are not directly related to biosafety in its technical decision. On the other hand, the separation leaves the market, the regulatory agencies or the CNBS with the economic, social, political or religious decisions.

Transparency and other issues of risk communication must be thoroughly assessed and the success of a commercial application for a new GM insect will strongly depend on how the different stakeholders will access the information. Reeves, et al. [11] suggest a checklist for assessing the scientific quality of approvals for un-caged field trials that may guide the risk analyst to help risk assessors and delineate an adequate strategy to overcome public mistrust and opposition, both in field trials and in commercial releases. The list, however, suggests the requirement to provide biological materials to independent investigators and some other measures that can hinder a final assessment, instead of producing a more transparent technical opinion.

Moreover, the technology developer must act accordingly and should bring to the public the largest possible set of useful information in accessible language to gather the public cooperation and reduce mistrust. This was effectively done in Brazil during the GM *A. aegypti* field releases back in 2011–2012 and is currently unfolding as a successful strategy in the mass releases for *A. aegypti* control in Piracicaba, Brazil [12], leading to a reduction in opposition and a to a stronger engagement of stakeholders.

## Gene drives for the control of insect populations under the Brazilian GMO regulatory framework and the new breeding technologies (NBTs)

The recent discovery of new ways to efficiently edit genomes of many organisms, including insects, coupled with the insertion of gene drives in the newly edited genomes, opened a whole new field of applications for both genome editing and gene drives [13–15]. Vector and pest control is certainly one of the most appealing applications of gene drives, both for population reduction (or eradication) and population control [13, 16–19].

Gene drives, however, are such powerful tools that they immediately raised concerns among a wide range of stakeholders, including scientists, activists, politicians and health managers [19–21]. The public perception is relatively polarized against the use of gene drives in many countries and even among scientists the opinions vary widely. Once again opposition to GMOs has taken the lead in this debate on many forums, leading to a collective fear that can be reversed as knowledge about technology becomes more comprehensive and available.

For risk assessors, however, gene drives can be considered as genes having an increased non-Mendelian transmission rate, usually approaching 100%. All other aspects are similar to regular genes. Indeed, using the regular approach for GMO risk assessment commented above, a group of scientists and regulators came recently to the conclusion that gene drives for population substitution or population reduction, when applied to *Anopheles gambiae* in an African environment, do not pose important risks either to the environment or to human health [21]. A similar assessment may be made by CTNBio in the near future for gene drive insects, as its normatives and procedures are in close agreement with those of other official regulatory agencies and with international organizations, as the Center for Environment Risk Assessment.

The 2005 Brazilian Biosafety law defines what qualifies as a GMO. As many other former legislations, the Brazilian law and its decree regulate genetic engineering (GE) products, which should contain a transgenic DNA either in its genome or in extrachromosomal plasmid or in an organelle. Although the GE definition is rather ample, encompassing most new techniques, the constraint imposed by the presence of a new transgenic DNA could exclude certain products from CTNBio’s assessment, such as those created by genome editing, potentially generating a limbo for many new products. In fact, there seems to be a consensus among Brazilian scientists that not all products derived from the new breeding technologies (NBTs) should be regulated and assessed before being released, especially if they do not contain “foreign” DNA or RNA. CTNBio can, by law, decide how to resolve omissions in its normative resolutions - and has often acted accordingly - in the assessment of genetically modified organisms. Therefore, it has the necessary tools to reach and apply its internal consensus to NBTs in a consistently science-oriented manner.

## Conclusions

In conclusion, the authors would like to emphasize that the genesis of an efficient regulatory system naturally depends on a balance of forces between those interested in modern biotechnology and those opposed to it. If pro-biotech players are the determinants of the regulatory process, it will probably be based on risk assessment recommendations. If, on the other hand, the opinion of the opposition group prevails, socio-economic issues will be placed side-by-side in the risk assessment process, which confuses the risk assessor and leads to frequent deadlocks. However, irrespective of the many methodological approaches to GMO risk analysis, at the end of the day it is often political will that determines GMO regulatory processes [22].

## Abbreviations

CIBio: Internal biosafety committees
CNBS: National biosafety council
CTNBio: National biosafety technical committee
EMBRAPA: Brazilian agricultural research corporation
GE: Genetic engineering
GMO: Genetically modified organism
IBAMA: Institute of environment and natural resources
NBT: New breeding technology
